# Galectin-3 Plays a Role in Neuroinflammation in the Visual Pathway in Experimental Optic Neuritis

**DOI:** 10.3390/cells13070612

**Published:** 2024-03-31

**Authors:** Masako Funaki, Junko Nio-Kobayashi, Ryoji Suzuki, Yoshio Bando

**Affiliations:** 1 Department of Anatomy, Akita University Graduate School of Medicine, Akita 010-8543, Japan; 2 Department of Functional Glycobiology in Infectious Diseases, National Research Center for the Control and Prevention of Infectious Diseases, Nagasaki University, Nagasaki 852-8523, Japan

**Keywords:** galectin-3, glia, demyelination, EAE, optic neuritis

## Abstract

Multiple sclerosis (MS) is an inflammatory demyelinating disease of the central nervous system (CNS) featuring numerous neuropathologies, including optic neuritis (ON) in some patients. However, the molecular mechanisms of ON remain unknown. Galectins, β-galactoside-binding lectins, are involved in various pathophysiological processes. We previously showed that galectin-3 (gal-3) is associated with the pathogenesis of experimental autoimmune encephalomyelitis (EAE), an animal model of MS. In the current study, we investigated the expression of gal-3 in the visual pathway in EAE mice to clarify its role in the pathogenesis of ON. Immunohistochemical analysis revealed upregulation of gal-3 in the visual pathway of the EAE mice during the peak stage of the disease, compared with naïve and EAE mice during the chronic stage. Gal-3 was detected mainly in microglia/macrophages and astrocytes in the visual pathway in EAE mice. In addition, gal-3^+^/Iba-1^+^ cells, identified as phagocytic by immunostaining for cathepsin D, accumulated in demyelinating lesions in the visual pathway during the peak disease stage of EAE. Moreover, NLRP3 expression was detected in most gal-3^+^/Iba-1^+^ cells. These results strongly suggest that gal-3 regulates NLRP3 signaling in microglia/macrophages and neuroinflammatory demyelination in ON. In astrocytes, gal-3 was expressed from the peak to the chronic disease stages. Taken together, our findings suggest a critical role of gal-3 in the pathogenesis of ON. Thus, gal-3 in glial cells may serve as a potential therapeutic target for ON.

## 1. Introduction

Multiple sclerosis (MS) is an immune-mediated demyelinating disease of the central nervous system (CNS) [1,2]. Optic neuritis (ON) is an inflammatory demyelinating disease of the optic nerve that is strongly associated with MS. ON can be the first sign and most common symptom of MS [3]. Approximately 50% of people with MS develop ON. ON is also linked to neuromyelitis optica (NMO), also known as Devic’s disease [4]. The most common cause of ON is inflammatory demyelination of the optic nerve [5]. However, the mechanisms underlying the immune-mediated inflammatory attack are unclear.

Experimental autoimmune encephalomyelitis (EAE) is the most widely used experimental animal model of human inflammatory demyelinating diseases such as MS [6,7,8]. Because the pathogeneses of EAE and MS share similar features and are related to ON and NMO, EAE can be used as a disease model of ON [9,10]. Indeed, accumulating evidence suggests that EAE induces significant retinal ganglion cell (RGC) loss [11,12,13]. Interestingly, Quinn et al. reported RGC loss in the EAE model, occurring more than 25 days after the immunization to induce EAE [11]. These observations indicate that ON in the EAE model develops gradually, along with the relatively slow degeneration of RGCs. However, the molecular mechanisms of ON in EAE and human MS remain unknown.

Galectins, a family of β-galactosidase lectins, consisting of 15 members in mammals, bind β-linked galactose, including *N*-acetyllactosamine (LacNAc), consisting of Gal β1-3/4 linked to *N*-acetylglucosamine (GlcNAc), in cell surface glycoproteins and glycolipids, as well as proteoglycans in the extracellular matrix. Among the family members, galectin-3 (gal-3) is expressed in the CNS and regulates various physiological and pathological events [14]. Autoantibodies against gal-3 have been reported to be present in sera of patients with secondary progressive MS and contribute to the persistent blood–brain barrier (BBB) breakdown [15]. The role of gal-3 in demyelinating animal models is controversial [16]. However, gal-3 knockout mice develop a significantly milder EAE and exhibit less inflammatory infiltrates in the spinal cord and reduced demyelination during EAE [17]. In addition, pro-inflammatory cytokines such as TNF-α are not increased in gal-3 knockout mice with cuprizone-induced CNS demyelination. In the streptozotocin (STZ)-induced diabetic model, gal-3 knockout mice show reduced neuroinflammation and fewer Iba-1-positive cells in the optic nerve [18]. Furthermore, our previous report demonstrated that gal-3 is expressed on activated microglia in the spinal cord, infiltrating macrophages in the spinal parenchyma and pia mater and Schwann cells in the ventral nerve roots in EAE [16]. Gal-3 is also expressed in F4/80(+) macrophages/microglia during EAE [19,20]. While the accumulating evidence suggests that gal-3 is involved in neuroinflammation in the pathogeneses of EAE and ON, the association of gal-3 with EAE-induced ON remains unclear.

In the current study, we investigated the expression and distribution of gal-3 in the visual pathway during EAE to clarify its role in the pathogenesis of ON.

## 2. Material and Methods

### 2.1. Animals

In all experiments, we utilized adult female C57BL/6J strain mice (6–8 weeks old) acquired from SLC in Shizuoka, Japan. The experimental procedures were conducted with approval from the Institutional Committee for Experimental Animals (#a-1-0294, Akita University, Japan).

### 2.2. EAE Induction

EAE induction followed a previously described protocol [6,21,22]. Briefly, myelin protein peptide, including MOG_35-55_ (MEVGWYRSPFSRVVHLYRNGK), was synthesized by Scrum in Tokyo, Japan. Mice were subcutaneously immunized in the flank with an emulsion containing 75 μL of complete Freund’s adjuvant (Sigma-Aldrich, St. Louis, MO, USA) and 0.4 mg of heat-inactivated Mycobacterium tuberculosis (H37Ra; BD/Difco Laboratories, Franklin Lakes, NJ, USA). Additionally, each animal received 200 ng of pertussis toxin (Sigma-Aldrich) intraperitoneally on days 0 and 2 post-immunization. EAE clinical scores were recorded as previously described [6,21,22].

### 2.3. Immunohistochemistry

Animals, deeply anesthetized, were sacrificed and perfused with normal saline followed by 4% paraformaldehyde (PFA) in 0.1 M phosphate buffer (PB) [6,9,21,22,23]. The brain, including the optic nerve, optic chiasm, and the optic tract, was removed and immersed in 30% sucrose in PBS for 1–2 days. After freezing in OCT medium, tissues were sectioned into 20 μm slices using a cryostat (Leica 1950; Leica, Germany) and stored at −30 °C until use. In some experiments, sections were stained with FluoroMyelin Red (FM; a lipophilic stain for compact myelin, ThermoFisher Scientific, Eugene, OR, USA) to assess demyelination as described previously [6,23]. The demyelinated lesion is shown as the FluoroMyelin-negative area in the visual pathway. The demyelinated area (% demyelination) in each section was quantified using ImageJ software (version 1.52-1.54, NIH, Bethesda, MD, USA) [6,21].

For immunohistochemistry, sections were pretreated with blocking solution (5% albumin, 2% normal goat serum and 0.3% tritonX-100) for 30 min at room temperature (RT) [6,9,21,22,23]. Sections were then incubated with primary antibodies overnight at 4 °C. The following antibodies were used as primary antibodies: anti-galectin-3 antibody (1:200; Santacruz Biotechnologies, Dallas, TX, USA), anti-Iba-1 polyclonal antibody (1:2000; WAKO, Osaka, Japan), anti-Iba-1 monoclonal antibody (1:200; WAKO), anti-GFAP antibody (1:2000; Sigma-Aldrich), anti-cathepsin D antibody (1:200; R&D Systems, Minneapolis, MN, USA), anti-NLR family, pyrin domain containing 3 (NLRP3) antibody (1:1000; Adipogen Life Sciences, Füllinsdorf, Switzerland). After washing with PBS, the sections were incubated with corresponding secondary antibodies (Alexa Fluor; Thermo Fisher Scientific) for 90 min at RT. For most double immunofluorescence experiments, DAPI-Fluoromount-G (SouthernBiotech, Birmingham, AL, USA) was used as a mounting medium. The sections were then analyzed with a confocal laser microscope (LSM-780 and LSM-510; Carl Zeiss, Oberkochen, Germany) or a fluorescence microscope (BZ-X800; Keyence, Tokyo, Japan). Cell counting is commonly performed using HE staining, but in this study, we introduced a fluorescence staining (DAPI staining) method in addition to the traditional HE staining for cell quantification. It is important to emphasize that the DAPI staining method specifically labels cell nuclei, providing a reliable and comprehensive means to assess the presence of cells. Inflammatory foci were characterized by the detection of more than 20 inflammatory cells within the perivascular area surrounding a specified blood vessel [6,21,24,25,26].

### 2.4. Immunoblotting

Animals, deeply anesthetized, were sacrificed and perfused with normal saline [6,9,21,22,23]. Subsequently, the optic nerve, chiasm, and tract were dissected and lysed in cell lysis buffer (Cell lytic M lysis buffer; Sigma-Aldrich). After determining the protein concentration using the DC protein assay kit (Bio-Rad Laboratories, Hercules, CA, USA), 6 µg of protein extract underwent separation by 15% SDS-PAGE, followed by transfer to a PVDF membrane (Millipore, Darmstadt, Germany). Immunostaining was performed using either anti-gal-3 antibody (1:500; Santacruz Biotechnologies) or anti-GAPDH antibody (1:5000; Sigma-Aldrich). HRP-conjugated secondary antibodies (GE Healthcare Systems, Sunnyvale, CA, USA) were applied, and the chemiluminescence assay (ECL) from GE Healthcare Systems was used. Chemiluminescence signals were detected using a Luminoimage Analyzer system (ChemiDoc Touch MP; Bio-Rad).

### 2.5. Statistical Analysis

Unless otherwise specified, each experimental group consisted of five to nine animals. Histochemical analysis utilized randomly chosen sections from more than three animals per group, analyzed using ImageJ software (NIH). The results are presented as mean ± SEM. Unpaired *t*-tests were employed for pairwise comparisons, and one-way ANOVA followed by Bonferroni tests was used for multiple comparisons. Statistical significance was set at *p* < 0.05 [6,9,21,22,23].

## 3. Results

### 3.1. EAE Induces Inflammation in the Visual Pathway

**Figure 1:**
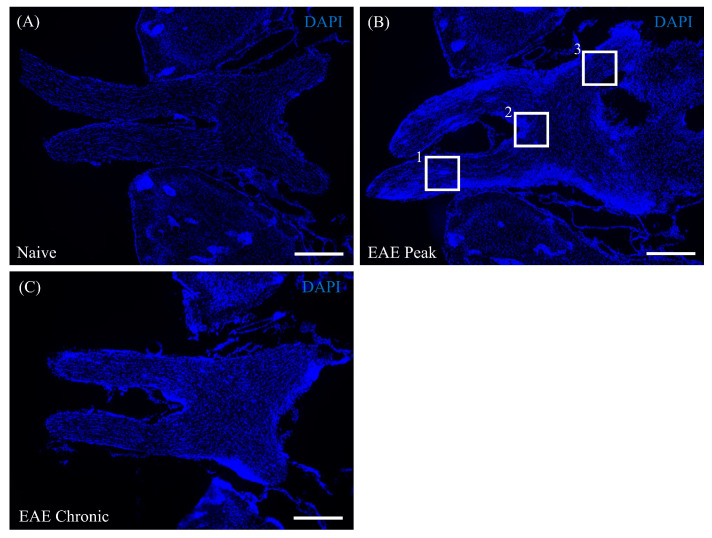
ON resulting from MOG-induced EAE. Representative sections of the visual pathway including the optic nerve, optic chiasm, and optic tract in naïve (**A**) and EAE mice at the peak disease stage (**B**) and chronic stage (**C**). Each section was stained with DAPI (a marker of nuclei, blue) (*N* = 6). The insets show the optic nerve (1), optic chiasm (2), and optic tract (3), respectively. Note that EAE was mainly associated with meningeal inflammation of the pia mater during the peak disease stage in the visual pathway compared with naïve mice. Scale bar = 500 μm.

Female C57BL/6J mice (6–8 weeks old) were immunized with MOG_35-55_ peptide to induce EAE. These mice showed typical symptoms, such as hindlimb paralysis, and were likely to reach the acute phase (peak disease) at 16–20 days and the chronic phase at 38–41 days after MOG immunization [6,21,22]. Compared with naive mice (Figure 1A), DAPI staining clearly demonstrated that the mice with MOG-induced EAE developed inflammation during the peak disease stage in the visual pathway, including the optic nerve (Figure 1B, square 1), optic chiasm (Figure 1B, square 2), and optic tract (Figure 1B, square 3). In addition, the mice showed meningeal inflammation of the pia mater in the visual pathway (Figure 1B). Inflammation in the chronic phase tended to be less than in the acute phase (Figure 1C). This finding is suggestive of gliosis and infiltration of peripheral immune cells into the visual pathway, resulting in meningeal inflammation with demyelination and neurodegeneration, characteristic of ON.

### 3.2. Mice with MOG-Induced EAE Show Upregulation of Gal-3 in Microglia and Macrophages in the Visual Pathway

**Figure 2:**
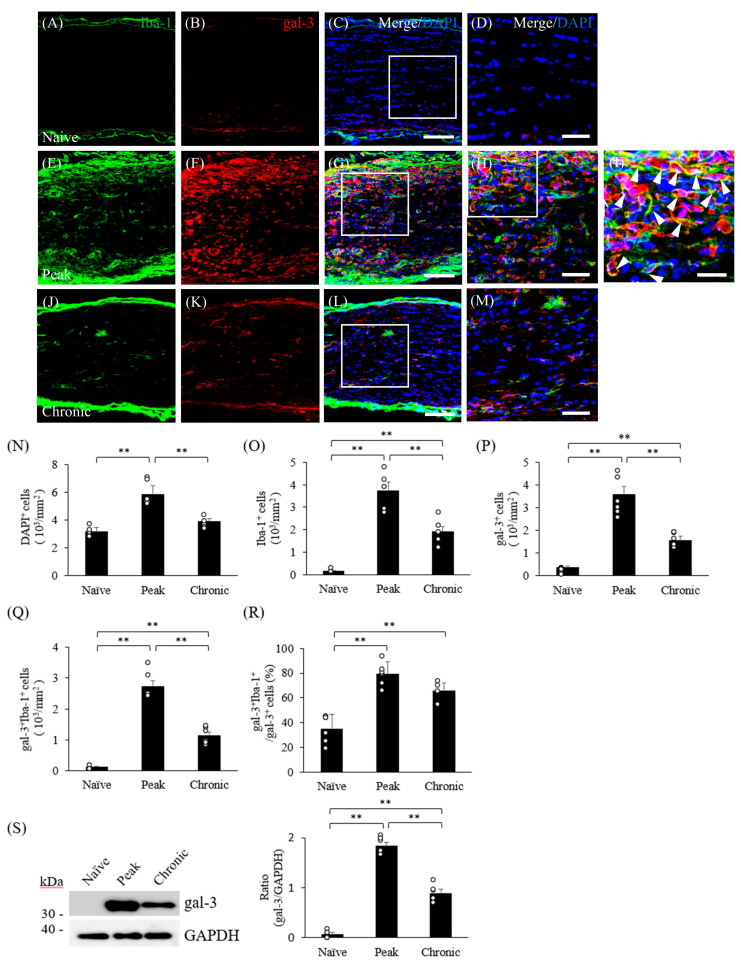
Microglial expression of gal-3 in ON associated with MOG-induced EAE. Naïve (**A**–**D**) and EAE mice were perfused at either the peak (**E**–**I**) or chronic (**J**–**M**) disease stage after MOG immunization. Frozen sections of the optic nerve to optic tract were stained with anti-Iba-1 antibody (a marker of microglia; (**A**,**E**,**J**), green) and anti-gal-3 antibody ((**B**,**F**,**K**), red). DAPI staining (blue) was also performed. Merged images are also shown (**C**,**D**,**G**–**I**,**L**,**M**). High-magnification images of the regions in the insets (**C**,**G**,**L**) are also shown (**D**,**H**,**M**). High-magnification image of the inset in (**H**) is shown in (**I**). Galectin-3 is co-expressed in Iba-1^+^ cells (white arrows). (**N**–**S**) Quantitative analysis. The numbers of DAPI^+^ cells (**N**), Iba-1^+^ cells (**O**), gal-3^+^ cells (**P**), gal-3^+^Iba-1^+^ cells (**Q**), and gal-3^+^Iba-1^+^ cells/gal-3^+^ cells (**R**) in the optic nerve of naïve and EAE mice. Six mice per group were examined. (**S**) Immunoblot analysis showing expression of gal-3 in the optic nerve from naïve and EAE mice. GAPDH was used as a loading control. Representative data are shown. Ratio of Galectin-3/GAPDH (*N* = 5). Scale bars: (**C**,**G**,**L**): 100 μm; (**D**,**H**,**M**): 50 μm; (**I**): 25 μm. ** *p* < 0.01.

Our previous study demonstrated that the adaptive transfer model of EAE induces gal-3 expression in activated microglia/macrophages in the white matter of the spinal cord [16]. Here, we examined gal-3 expression during disease progression in the visual pathway in mice with MOG-induced ON. Anti-Iba-1 antibody was used as a marker of both microglia and infiltrating peripheral macrophages in the CNS. Compared with naïve mice (Figure 2A,O), the number of Iba-1^+^ microglia/macrophages was dramatically increased in the optic nerve during the peak disease (Figure 2E,O). Consistent with these observations, naïve mice had negligible expression of gal-3 in the optic nerve (Figure 2B,P). The number of gal-3^+^ cells increased during the peak disease stage (Figure 2F,P), compared with naïve mice (Figure 2B,P). Furthermore, about 80% of Iba-1^+^ microglia/macrophages expressed gal-3 (Figure 2G–I,Q,R). At 42 days after MOG immunization, which is the chronic disease stage (Figure 2J–M), about 65% of Iba-1^+^ microglia/macrophages expressed gal-3 (Figure 2Q,R). As observed in Figure 1B, the number of DAPI^+^ cells in the optic nerve was significantly increased at the peak disease stage (Figure 2N). Immunoblot analysis also showed that the expression level of gal-3 was significantly increased during the peak disease compared with naïve mice (Figure 2S). These results indicate that the expression of gal-3 in microglia/macrophages is associated with the pathogenesis of ON caused by EAE-induced neuroinflammation. Similar results were obtained for the optic chiasm (Figure 1B, square 2 and Figure S1) and the optic tract (Figure 1B, square 3 and Figure S2).

### 3.3. Astrocytic Expression of Gal-3 in the Optic Nerve during EAE

**Figure 3:**
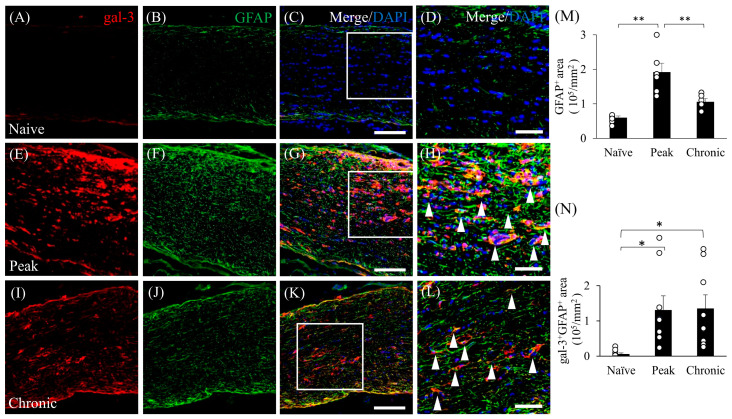
Astrocytic expression of gal-3 in ON caused by MOG-induced EAE. Frozen cross-sections were stained with anti-gal-3 antibody ((**A**,**E**,**I**); red) as well as anti-GFAP antibody (a marker of astrocytes, (**B**,**F**,**J**), green). Merged images with DAPI staining (blue) are shown in (**C**,**G**,**K**), respectively. High-magnification images of the insets in (**C**,**G**,**K**) are shown in (**D**,**H**,**L**), respectively. Galectin-3 is co-expressed in GFAP^+^ cells (white arrows). Representative data are shown. Quantitative analysis of GFAP^+^ area (**M**) (6 mice) and gal-3^+^/GFAP^+^ area (**N**) (9 mice). Scale bars: (**C**,**G**,**K**): 100 μm; (**D**,**H**,**L**): 50 µm. * *p* < 0.05, ** *p* < 0.01.

Astrocytes are also involved in the pathogenesis of EAE, and therefore, we next examined the expression of gal-3 in astrocytes. Consistent with previous reports in the EAE spinal cord [6,22], EAE caused accumulation of GFAP^+^ astrocytes in the optic nerve during the peak disease stage (Figure 3). While little expression of gal-3 in astrocytes was observed in naïve mice (Figure 3A), increased expression of gal-3 was observed at the peak and chronic stages of EAE-induced ON (Figure 3F,J). Consistent with this observation, immunohistochemical analysis showed increased gal-3 expression at the peak and chronic stages of EAE-induced ON (Figure 3E,I), compared with naïve mice (Figure 3A). Furthermore, the number of gal-3^+^/GFAP^+^ cells was significantly increased during the peak and chronic stages of EAE compared with naïve mice (Figure 3D,H,L–N).

### 3.4. Accumulation of Gal-3^+^ Cells in the Demyelinating Lesions in EAE

**Figure 4:**
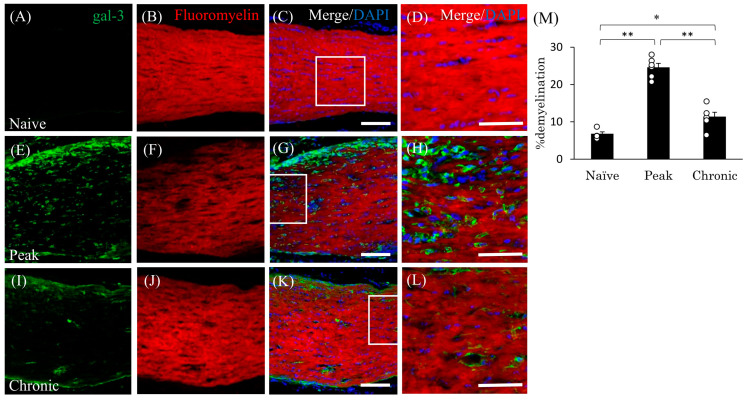
Involvement of gal-3^+^ cells in optic nerve demyelination in mice with EAE. Frozen cross-sections were stained with anti-gal-3 antibody ((**A**,**E**,**I**), green) as well as FluoroMyelin (a marker of myelin; (**B**,**F**,**J**), red). Merged images with DAPI (blue) are shown (**C**,**D**,**G**,**H**,**K**,**L**). High-magnification images of the insets in (**C**,**G**,**K**) are shown in (**D**,**H**,**L**), respectively. (**M**) Quantitative analysis of % demyelination (*N* = 6). Scale bars: (**C**,**G**,**K**): 100 µm; (**D**,**H**,**L**): 50 μm. * *p* < 0.05, ** *p* < 0.01.

**Figure 5:**
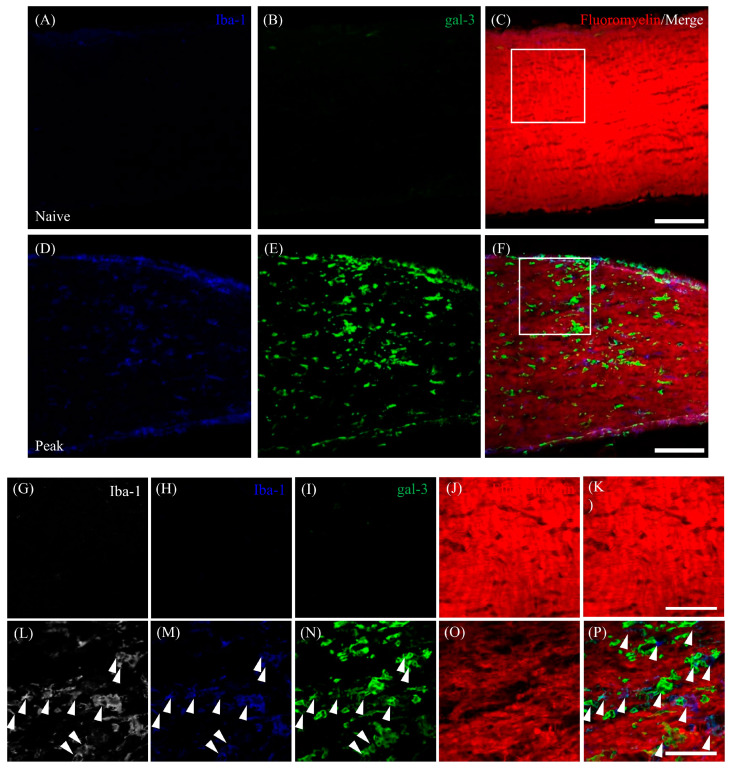
Association of gal-3^+^/Iba-1^+^ cells with demyelination in the EAE optic nerve. The frozen cross-sections were triple-stained with anti-Iba-1 antibody ((**A**,**D**,**H**,**M**), blue; (**G**,**L**), white), anti-gal-3 antibody ((**B**,**E**,**I**,**N**); green), and FluoroMyelin ((**J**,**O**); red). Merged images with FluoroMyelin are shown (**C**,**F**,**K**,**P**). (**G**–**P**) High-power images of the insets in (**C**,**F**) are also shown. Galectin-3^+^Iba-1^+^ cells are located in demyelinated lesions (white arrows). Six mice per group were examined. Representative data are shown. Scale bars: (**A**–**C**,**E**–**G**): 100 µm; (**D**,**H**): 50 μm.

To examine the involvement of gal-3-expressing cells in EAE-induced demyelination in the optic nerve, immunohistochemical analysis combined with FluoroMyelin staining was performed. Compared with naïve mice (Figure 4B), FluoroMyelin staining demonstrated that EAE mice showed demyelination in the optic nerve during the peak disease stage (Figure 4F,M). Remyelination was observed at the chronic stage (Figure 4J,M). As shown in Figure 4E–H, immunohistochemistry clearly demonstrated the accumulation of gal-3-expressing cells in the demyelinating lesion in MOG-induced ON (Figure 4E,G,H). Furthermore, gal-3^+^/Iba-1^+^ microglia/macrophages were detected in the demyelinating lesions during the peak disease stage (Figure 5F,P). These results suggest that gal-3^+^ microglia/macrophages are involved in optic nerve demyelination in mice with MOG-induced EAE.

### 3.5. Expression of Gal-3 in Cathepsin D-Expressing Activated Microglia/Macrophages

**Figure 6:**
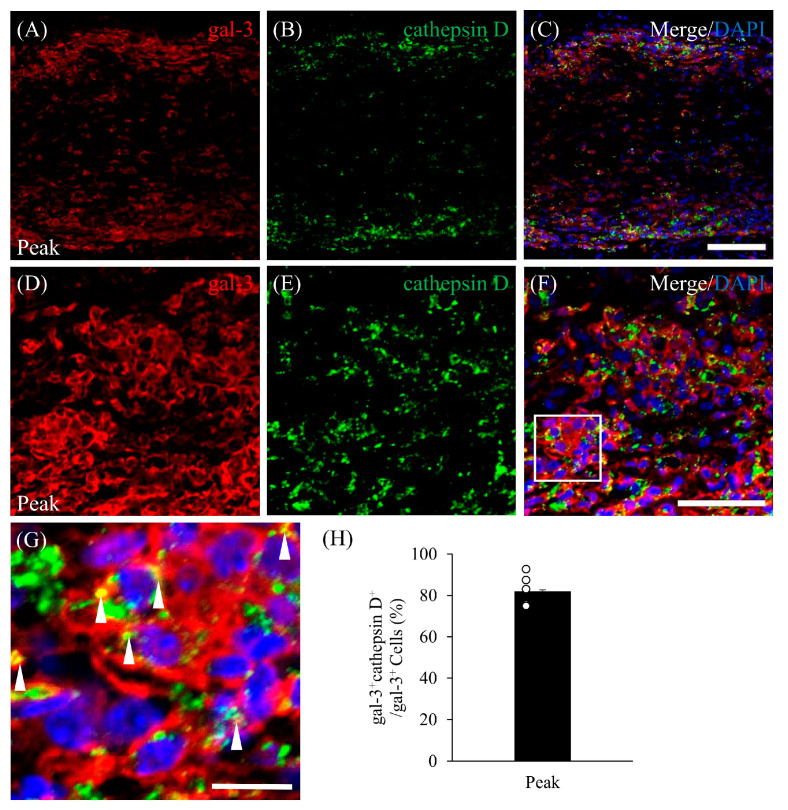
Co-expression of cathepsin D and gal-3 in the EAE optic nerve. Frozen cross-sections were stained with anti-gal-3 antibody ((**A**,**D**), red) as well as anti-cathepsin D antibody (a marker of phagocytotic microglia/macrophages; (**B**,**E**), green). Merged images with DAPI staining (blue) are shown (**C**,**F**,**G**). High-magnification image of inset in F is shown in (**G**). White arrowheads represent gal-3^+^ cells expressing cathepsin D. Representative data are shown. (**H**) Quantitative analysis of cathepsin D^+^/gal-3^+^ cells (*N* = 5). Note that about 80% of gal-3^+^ cells expressed cathepsin D in the EAE optic nerve at the peak disease stage. Scale bars: (**A**–**C**): 100 μm; (**D**–**F**): 50 µm; (**G**): 25 µm.

To elucidate the pathophysiological function of gal-3-expressing microglia/macrophages in the demyelinating lesions in MOG-induced ON, immunohistochemistry with anti-cathepsin D antibody was performed [16]. Immunohistochemistry revealed that about 80% of gal-3^+^ cells expressed cathepsin D (Figure 6C,H), suggesting that it plays a role in phagocytosis by microglia/macrophages in the lesions at the peak (Figure 6A–C) and chronic (Figure 6D–G) stages of the disease. Cathepsin D^+^ cells accumulated in the inflammatory sites of the meninges during the peak disease stage of MOG-induced ON (Figure 6C,F,G).

### 3.6. Inflammasome Activation Upregulates Gal-3 in Microglia/Macrophages in Mice with EAE

**Figure 7:**
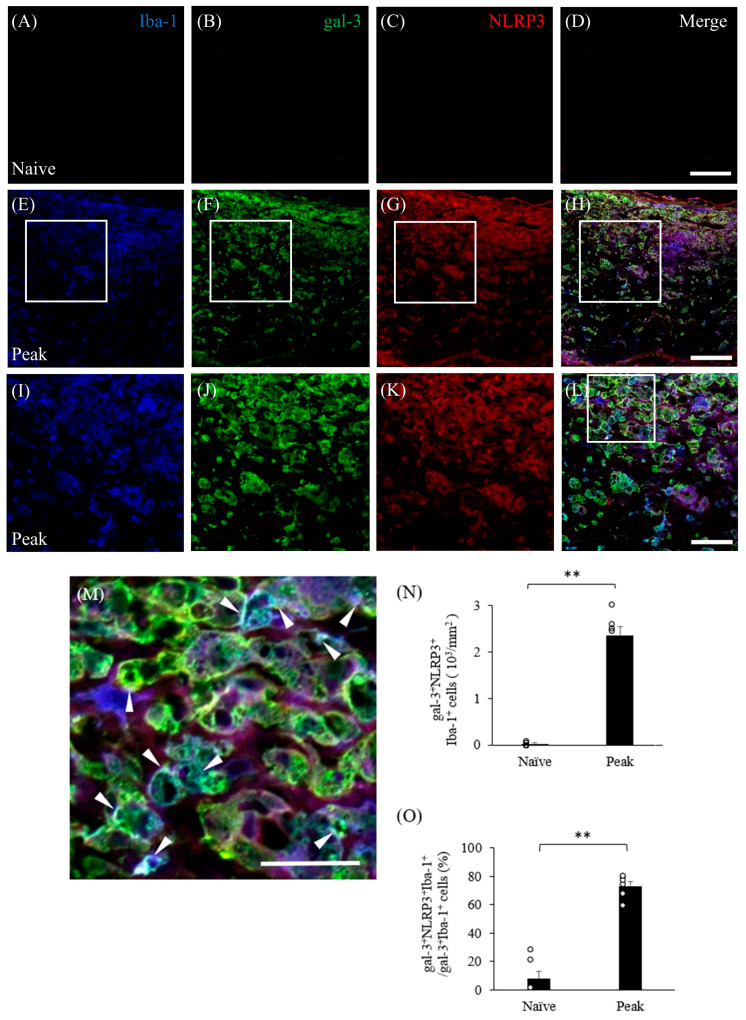
Expression of NLRP3 in gal-3^+^/Iba-1^+^ cells in the optic nerve of mice with EAE. Frozen cross-sections from naïve mice (**A**–**D**) and EAE mice at peak disease (**E**–**L**) were triple-stained with anti-Iba-1 antibody ((**A**,**E**,**I**), blue), anti-gal-3 antibody ((**B**,**F**,**J**), green), and NLRP3 (marker for inflammasomes; (**C**,**G**,**K**), red). Merged images are shown (**D**,**H**,**L**,**M**). High-magnification images ((**H**), inset) are also shown (**I**–**L**). (**M**) High-magnification image of the inset in (**L**). Arrowheads show gal-3^+^/Iba-1^+^/NLRP3^+^ cells in the optic nerve of EAE mice. Representative data are shown. Quantitative analysis of the number of gal-3^+^/Iba-1^+^/NLRP3^+^ cells in the optic nerve (**N**) and the ratio of gal-3^+^/Iba-1^+^/NLRP3^+^ cells to gal-3^+^/Iba-1^+^ cells in the optic nerve (**O**). Six mice per group were examined. Scale bars: (**A**–**D**): 100 μm; (**E**–**H**): 50 µm; (**I**–**M**): 25 µm; (**L**): 12.5 µm ** *p* < 0.01.

**Figure 8:**
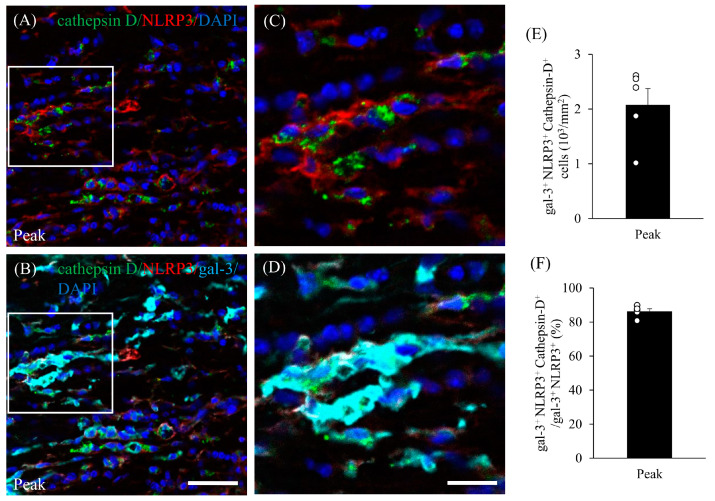
Expression of gal-3 in cathepsin D^+^/NLRP3^+^ cells in the optic nerve of mice with EAE. (**A**,**C**) Frozen cross-sections of the optic nerve from EAE mice at peak disease were stained with anti-cathepsin D antibody (green), anti-NLRP3 antibody (red), and DAPI (blue). High-magnification image of inset in (**A**) is shown in (**C**). (**B**,**D**). Optic nerve sections from EAE mice at peak disease were also stained with anti-cathepsin D antibody (green), anti-NLRP3 antibody (red), anti-gal-3 antibody (cyan), and DAPI (blue). High-magnification image of inset in (**B**) is shown in (**D**). Quantitative analysis of the number of gal-3^+^/NLRP3^+^/cathepsin D^+^ cells in the optic nerve (**E**) and the ratio of gal-3^+^/NLRP3^+^/cathepsin D^+^ cells to gal-3^+^/NLRP3^+^ cells in the optic nerve (**F**). Five mice per group were examined. Scale bars: (**A**,**B**): 50 μm; (**C**,**D**): 25 μm.

EAE induces gliosis and the infiltration of peripheral inflammatory cells into the CNS, followed by inflammatory demyelination. We therefore examined whether the inflammasome is activated in gal-3^+^ microglia/macrophages during the peak disease stage. Immunohistochemistry demonstrated that gal-3^+^/Iba-1^+^ activated microglia/macrophages also expressed NLRP3, which is a marker of inflammasomes (Figure 7). NLRP3 on gal-3^+^/Iba-1^+^ cells was also significantly induced in the inflammatory sites of the optic nerve at the peak disease stage (Figure 7H,L–N). In addition, about 80% of gal-3^+^/Iba-1^+^ cells expressed NLRP3 (Figure 7O). These observations suggest that gal-3 plays a role in inflammasome-related NLRP3 signaling in microglia/macrophages in MOG-induced ON. To assess the function of gal-3^+^/NLRP3^+^/Iba-1^+^ cells during MOG-induced ON, immunohistochemistry with anti-cathepsin D antibody was performed. Cathepsin D was detected in most gal-3^+^/NLRP3^+^ cells during the peak disease stage, indicating that gal-3^+^/NLRP3^+^/Iba-1^+^ cells have phagocytotic properties at the inflammatory sites of the meninges in the optic nerve in MOG-induced ON (Figure 8).

## 4. Discussion

Although the clinical importance of encephalomyelitis in ON is well-documented [10], the molecular mechanisms of ON remain unclear. In the current study, we found that gal-3 was expressed mainly in activated microglia/macrophages in the visual pathway, including the optic nerve, the optic chiasm, and the optic tract, in MOG-induced ON. These results suggest that gal-3 plays an important role in the pathogenesis of ON in MOG-induced EAE.

MOG-induced EAE, a widely used animal model of MS, is used mainly for investigating the neuropathological changes in the spinal cord because the animals develop severe spinal neuroinflammation followed by paralysis of the hindlimbs. However, optic nerves express higher levels of MOG than the spinal cord [13], and 30% of MOG-specific TCR transgenic mice spontaneously develop ON [27]. In addition, studies show that MOG-induced EAE can cause ON [12,13,28,29]. Furthermore, infiltration of peripheral immune cells into the optic nerve in EAE has been reported by H&E staining [11,29]. In the current study, we aimed to clarify the pathogenesis of ON in MOG-induced EAE.

We found here that mice with MOG-induced EAE develop ON 16–20 days after MOG immunization, as previously reported [11,29]. Similar to the pathological changes in the visual pathway in human neuromyelitis optica spectrum disorder (NMOSD) [30], C57BL/6J mice showed accumulation of inflammatory cells in the bilateral optic nerves, the optic chiasm, and the bilateral optic tracts during EAE development. The pathological features in EAE-induced ON were similar in all lesions, including the optic nerve, the optic chiasm, and the optic tracts in the visual pathway. Our findings show that the optic nerve undergoes striking pathological changes in EAE-induced ON. Although we did not examine the pathological changes in retinal ganglion cells (RGCs), a previous report showed that RGC loss occurs more than 25 days after immunization to induce EAE [11], strongly suggesting that RGC loss in the retina occurs by progressive anterograde degeneration.

Accumulation of inflammatory cells was observed along the meninges in the optic nerve, suggesting that microvascular changes promote the infiltration of immune cells into the visual pathway. MS is characterized by perivascular infiltration of immune cells and macrophages [31]. Here, we found increased numbers of Iba-1^+^ microglia/macrophages and GFAP^+^ astrocytes at inflammatory sites, including the meninges in the EAE optic nerve, indicating an association of microgliosis and astrogliosis with ON pathology. These findings suggest that meningeal inflammation may play a key role in the pathogenesis of EAE-induced ON. In addition, meningeal inflammation in MS and ON may induce microglial and astroglial phenotypic alterations, including the upregulation of gal-3, which might reflect disease progression. A previous report demonstrated that the inflammatory infiltrates in the meninges, especially the pia mater, produce a soluble factor that induces demyelination and axonal degeneration, either directly or indirectly through microglial activation [32].

The induction of gal-3 in microglia/macrophages likely plays an important role in neuroinflammation in the visual pathway in ON in EAE. While little to no gal-3 expression in the visual pathway, including the optic nerve, was observed in naïve mice, the number of gal-3-expressing Iba-1^+^ microglia/macrophages was significantly increased in the visual pathway in mice with EAE-induced ON, especially during the peak disease stage. Approximately 80% of gal-3^+^ cells in the EAE optic nerve were Iba-1^+^ macrophages/microglia at the peak disease. Gal-3^+^ microglia/macrophages in the optic nerve were observed mainly in the pia mater and surrounding tissue rather than the center of the optic nerve. This suggests that peripheral immune cells infiltrate into the optic nerve from the pia mater. Because activated microglia/macrophages are ameboid cells that have retracted their processes and have an enlarged soma [33], the gal-3^+^ microglia/macrophages in the EAE optic nerve likely represent activated cells during the peak disease stage. An important role of gal-3 in macrophage M1/M2 polarization and function has been reported [34].

The present findings suggest that gal-3^+^/NLRP3^+^/cathepsin D^+^ microglia/macrophages may play a key role in demyelination in EAE-associated ON during the peak disease stage. During the peak disease stage, gal-3^+^ microglia/macrophages expressed cathepsin D. Activated microglia/macrophages play an important role in neuroinflammation and phagocytosis in the CNS [35]. Cathepsin D is secreted by activated phagocytic microglia/macrophages [36,37]. Consistent with our previous report on the spinal cord in the acute phase of EAE [16], we found here that some gal-3^+^/Iba-1^+^ microglia/macrophages showed increased immunoreactivity for cathepsin D in the EAE optic nerve. In addition, most cathepsin D-expressing microglia/macrophages with a round shape were activated. By contrast, the cells with a ramified shape exhibited low expression of cathepsin D. Moreover, these microglia/macrophages accumulated in demyelinating lesions in the optic nerve.

Most gal-3^+^/Iba-1^+^ cells expressed NLRP3. NLRP3 is considered a key mediator of neuroinflammation by initiating the assembly of an inflammasome. The NLRP3 inflammasome triggers caspase-1 activation and IL-1β cytokine secretion, resulting in pyroptosis, recently identified as a programmed cell death regulated by the activation of the canonical caspase-1 signaling pathway [38]. The outcome of an M1 polarizing event is dependent on a number of factors, including the production of iNOS, reactive oxygen species (ROS), and activation of the NLRP3 inflammasome complex [39,40,41]. Emerging evidence suggests an important role of gal-3 in the activation of the NLRP3 inflammasome in M1-polarized macrophages. Interestingly, gal-3 promotes an inflammatory response by directly binding to NLRP3 [42]. These results suggest that gal-3 regulates NLRP3 signaling in microglia/macrophages, and that it may play a critical role in neuroinflammation and phagocytic activity during EAE. Therefore, NLRP3-expressing pro-inflammatory M1-polarized microglia/macrophages may serve as the first line of defense in EAE-induced ON. Indeed, the NLRP3 inflammasome has been implicated in EAE and MS in humans [43,44,45].

Gal-3 was still expressed on Iba-1^+^ microglia/macrophages at the chronic phase of EAE-induced ON. The clearance of abnormal proteins and protein debris such as myelin debris could be mediated by microglial cathepsin D [46]. Indeed, a previous study reported that following demyelination, microglia/macrophages remove myelin debris in the lesion [35]. Microglia/macrophages with phagocytotic properties may be M2-polarized and contribute to myelin debris clearance [47,48]. Moreover, gal-3 induces phagocytosis for myelin debris clearance in autoimmune demyelinating diseases [20,49,50]. Gal-3 is expressed in microglia that phagocytose damaged myelin, but not in microglia that do not do so [20,46]. Thus, unlike during the peak disease stage, gal-3^+^/NLRP3^+^/cathepsin D^+^ microglia/macrophages at the chronic phase might be M2-polarized to promote remyelination. Recent studies suggest that the categorization of microglial states is far too simplistic, and that the different microglial states are better represented as a spectrum of phenotypes [51,52,53]. Therefore, we did not classify these microglia/macrophages as M1 or M2 in this study. Gal-3 in microglia/macrophages might have different roles depending on the microenvironment. Further study is needed to clarify the functions of the protein in EAE and ON.

In the present study, we found that gal-3 was upregulated in astrocytes at the peak and chronic stages of EAE. The number of GFAP^+^ astrocytes was highest at the peak disease stage, and then decreased during the chronic stage, suggesting the induction of astrocytic gliosis. Similar to microglia/macrophages, negligible gal-3 expression was detected on astrocytes in the naïve ON. Sirko et al. reported that gal-3 is expressed on reactive proliferating astrocytes in the injured gray matter in the adult mouse cerebral cortex [54]. By contrast, in our previous study, astroglial gal-3 expression was not observed in the EAE spinal cord [16]. Thus, astrocytes might play differential roles in the optic nerve and spinal cord. Reactive astrocytes have both potentially beneficial and detrimental roles during remyelination. Indeed, these functions might be related to the specific phenotype of the astrocytes [55,56,57,58]. Astrocytes may promote/inhibit remyelination directly, but could also signal through microglia to promote remyelination [24,57,58,59]. Although the role of astrocytes during remyelination remains unclear, our results suggest that astrocytic gal-3 may also play a major role in remyelination in ON.

## 5. Conclusions

Our findings suggest that gal-3 has a key role in microglia/macrophages and astrocytes in neuroinflammation in ON. Activation of microglia/macrophages and astrocytes can be considered a double-edged sword, and the protective and harmful effects of gal-3 might depend on disease progression, stage, and microenvironmental factors. Gal-3 may have potential as a therapeutic target for ON.

## Data Availability

All data are available for sharing upon request.

## Supplementary Materials

The following supporting information can be downloaded at: https://www.mdpi.com/article/10.3390/cells13070612/s1, Figure S1: Expression of gal-3 in EAE optic chiasm; Figure S2: Expression of gal-3 in EAE optic tract.

## Abbreviations

BBB	blood–brain barrier
CNS	central nervous system
DAPI	4′,6-diamidino-2-phenylindole
EAE	experimental autoimmune encephalomyelitis
gal-3	galectin-3
GFAP	glial fibrillary acid protein
Iba-1	ionized calcium-binding adapter molecule 1
MBP	myelin basic protein
MOG	myelin oligodendrocyte glycoprotein
MS	multiple sclerosis
NLRP3	NLR family pyrin domain containing 3
NMO	neuromyelitis optica
ON	optic neuritis
OLs	oligodendrocytes
OPC	oligodendrocyte precursor cell
PBS	Phosphate-buffered saline
PFA	paraformaldehyde
