# Supplementary material for: Galectin-3 Plays a Role in Neuroinflammation in the Visual Pathway in Experimental Optic Neuritis
Source: Cells. 2024 Mar 31;13(7):612. doi: 10.3390/cells13070612 (PMC11011492; doi:10.3390/cells13070612)
Supplement: Supplementary file 1 [file cells-13-00612-s001.zip › cells-2864085-supplementary.pdf]

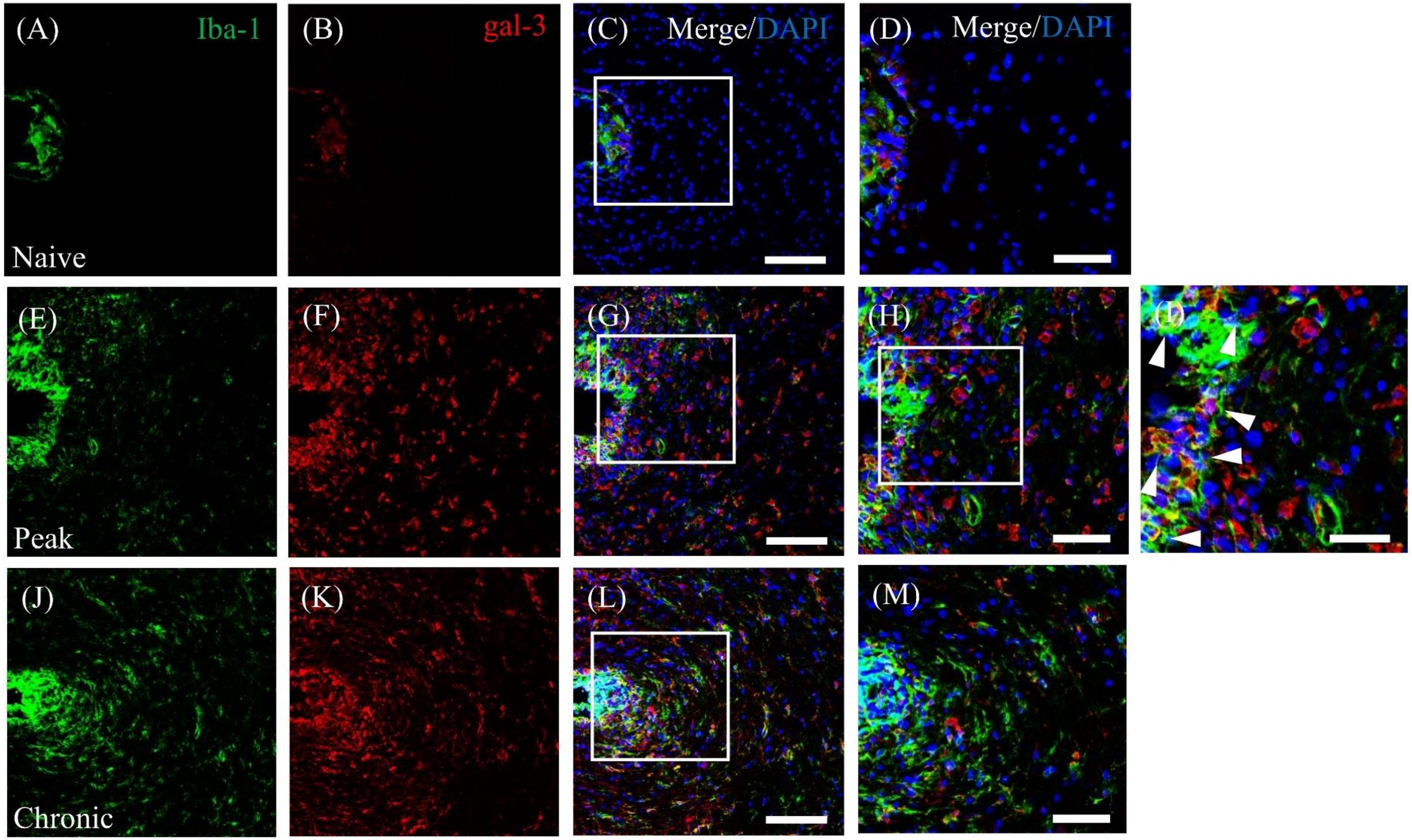

(A)
Iba-1
Naive
(B)
gal-3
(C)
Merge/DAPI
(D)
Merge/DAPI
(E)
Peak
(F)
(G)
(H)
(I)
(J)
Chronic
(K)
(L)
(M)

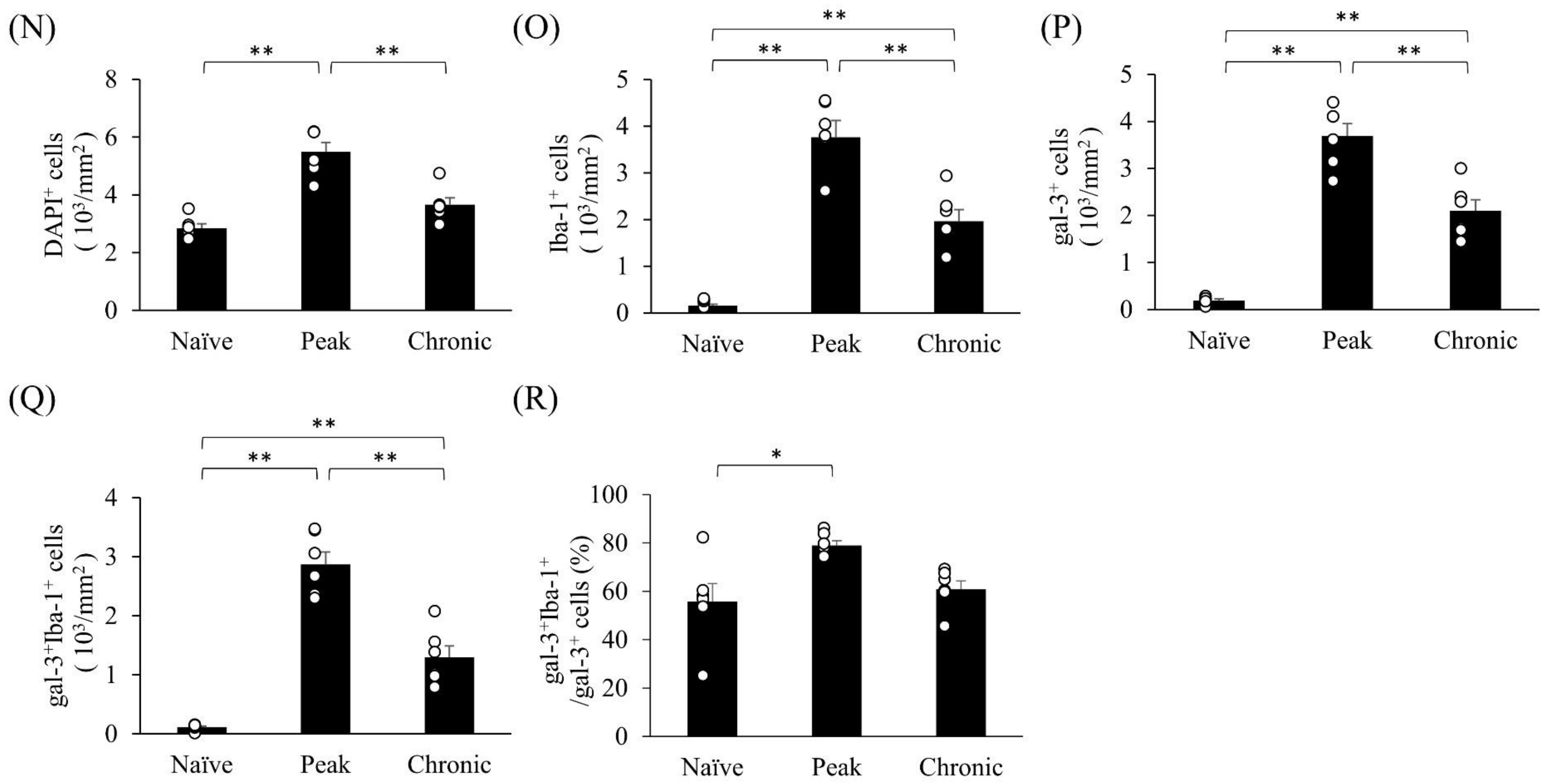

Figure S1. Expression of gal-3 in EAE optic chiasm. Naïve (A-D) and EAE mice were perfused at either the peak disease (E-I) or the chronic phase (J-M) after MOG immunization and optic nerve to optic tract with brain was taken out. Frozen sections were stained with anti-Iba-1 antibody (A, E, J, green) and anti-gal-3 antibody (B, F, K, red). DAPI staining (blue) was also performed. Merged images are also shown (C, D, G, H, I, L, M). Images with high-power magnification of each panel (C, G, L, inset) are shown, respectively (D, H, M). A High-power magnification of panel H (inset) is also shown in panel I. (N-R) Quantitative analysis were shown. The numbers of DAPI^{+} cells (N), Iba-1^{+} cells (O), gal-3^{+} cells (P), gal-3^{+}Iba-1^{+} cells (Q), and gal-3^{+}Iba-1^{+} cells/gal-3^{+} cells (R) in optic chiasm of naïve and EAE mice were shown. Six mice per group were examined. Scale

bars: C, G, L: 100 μm, D, H, M: 50 μm, I: 25 μm. *p<0.05, **p<0.01 are shown.

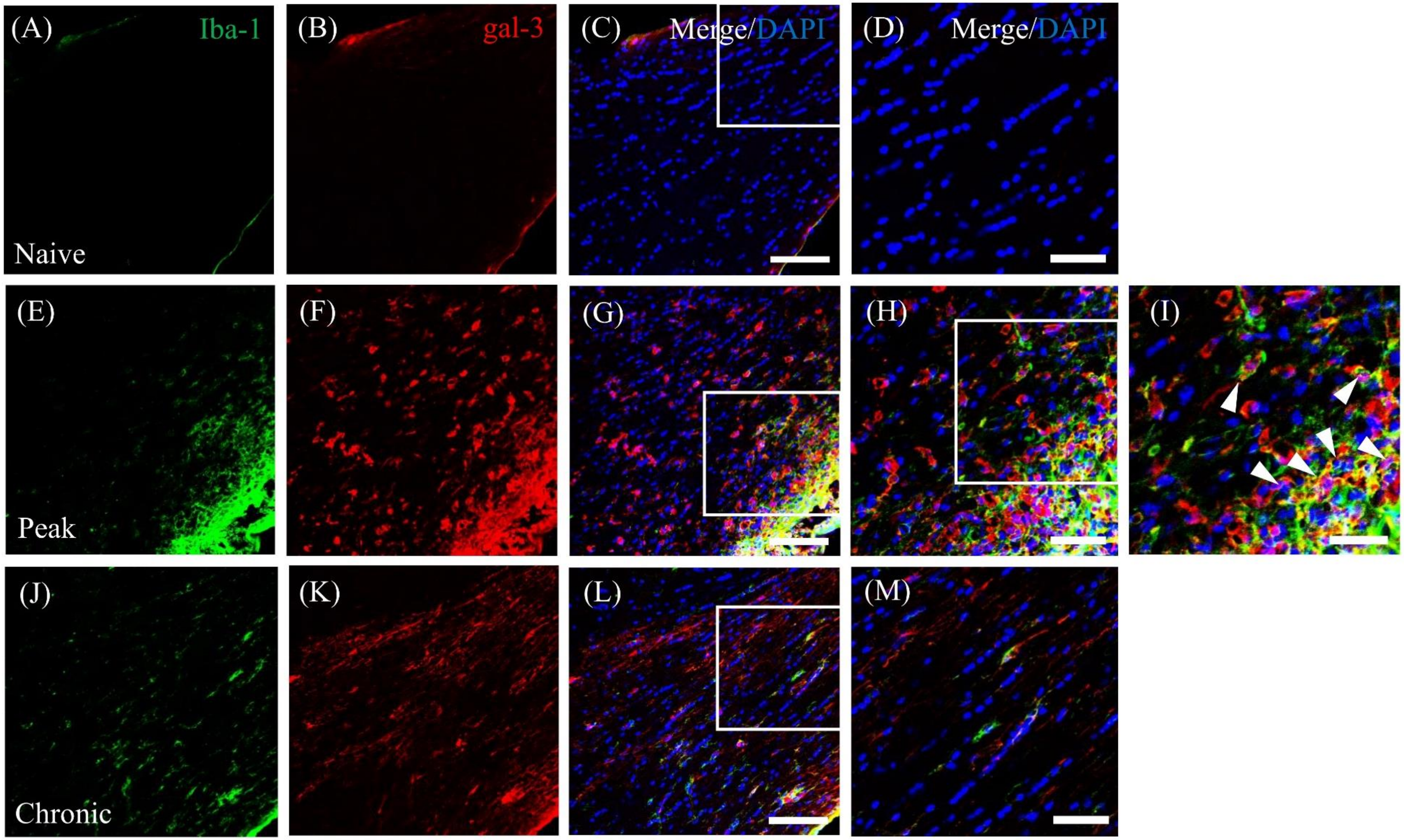

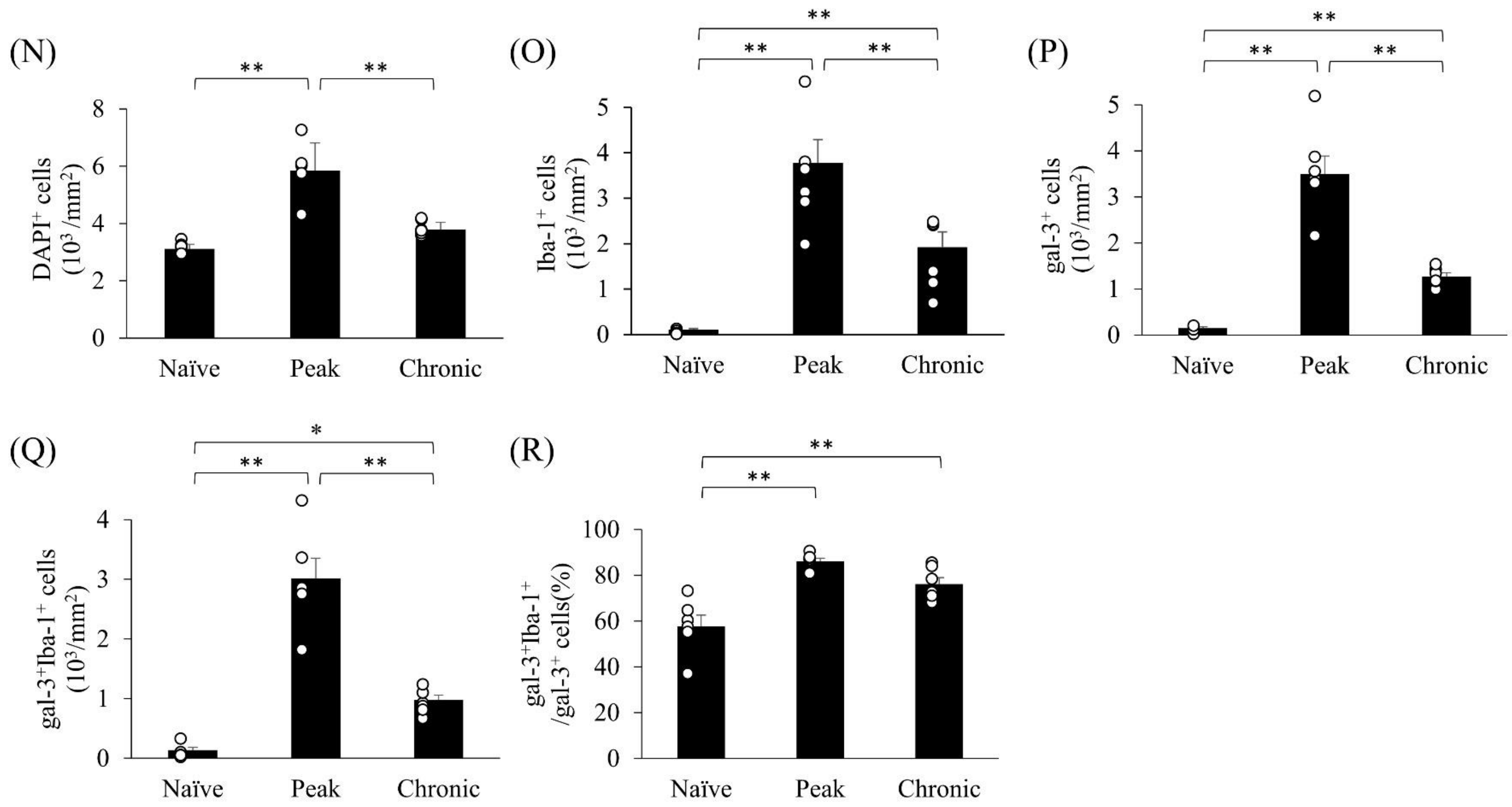

Figure.S2 Expression of gal-3 in EAE optic tract. Naïve (A-D) and EAE mice were perfused at either the peak disease (E-I) or the chronic phase (J-M) after MOG immunization and optic nerve to optic tract with brain was taken out. Frozen sections were stained with anti-Iba-1 antibody (A, E, J, green) and anti-gal-3 antibody (B, F, K, red). DAPI staining (blue) was also performed. Merged images are also shown (C, D, G, H, I, L, M). Images with high-power magnification of each panel (C, G, L, inset) are shown, respectively (D, H, M). A High-power magnification of panel H (inset) is also shown in panel I. (N-R) Quantitative analysis were shown. The numbers of DAPI$^+$ cells (N), Iba-1$^+$ cells (O), gal-3$^+$ cells (P), gal-3$^+$Iba-1$^+$ cells (Q), and gal-3$^+$Iba-1$^+$ cells/gal-3$^+$ cells (R) in optic tract of naïve and EAE mice were shown. Six mice per group were examined. Scale bars:

C, G, L: 100 μm, D, H, M: 50 μm, I: 25 μm. *p<0.05, **p<0.01 are shown.
